# Supplementary material for: The differential spatiotemporal expression pattern of shelterin genes throughout lifespan
Source: Aging (Albany NY). 2017 Apr 17;9(4):1219–28. doi: 10.18632/aging.101223 (PMC5425123; doi:10.18632/aging.101223)
Supplement: Supplementary file 1 [file aging-09-1219-s001.pdf]

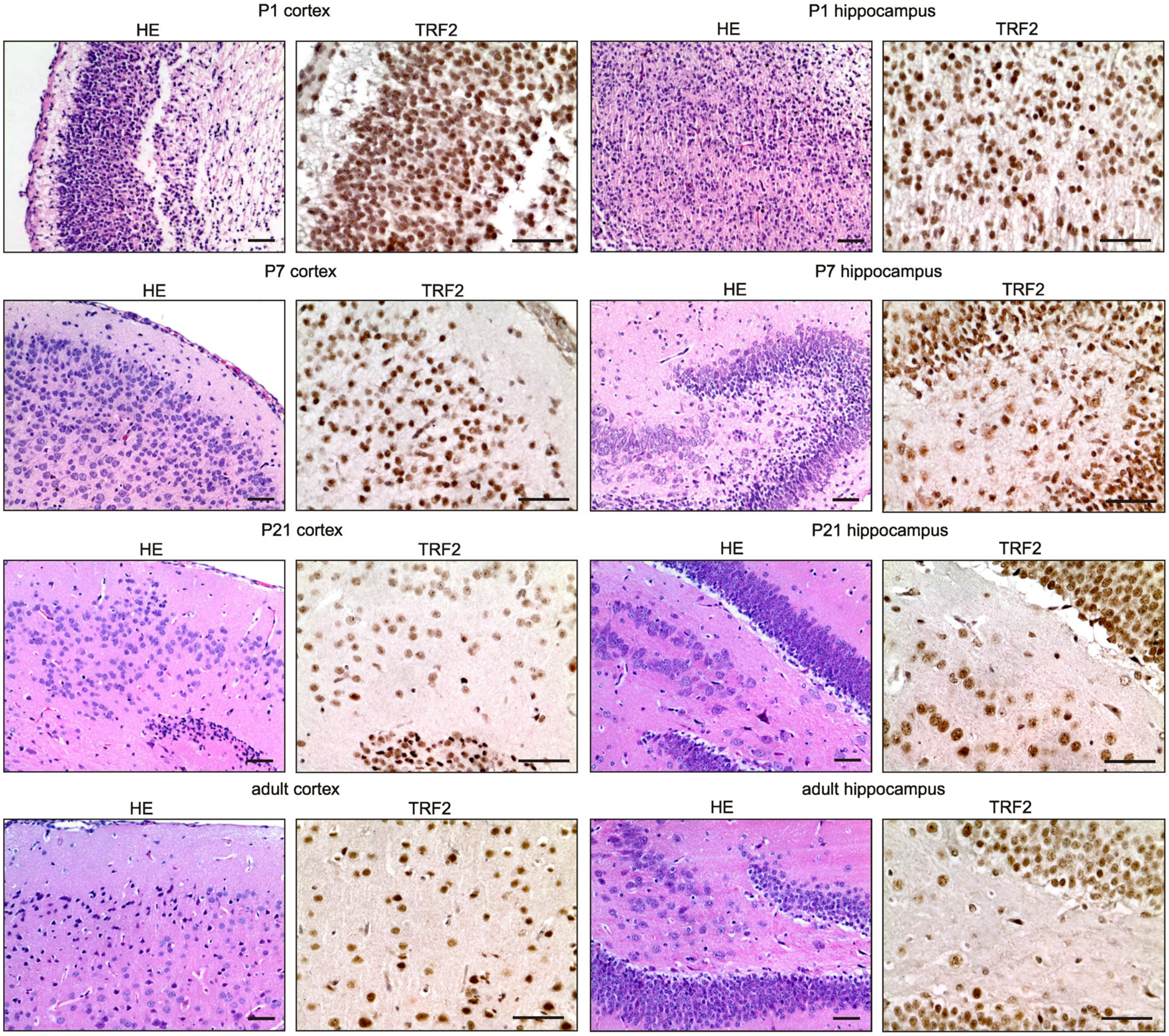

Figure S1 related to Figure 3. TERF2 continues to be highly expressed in neurons of the brain during adulthood representative photomicrographs of TERF2 immunostaining in the cortex and the hippocampal region of the brain (3,3' diaminobenzidine (DAB) substrate, brown, hematoxylin counterstaining) at different stages after birth. The left panels show hematoxylin-eosin (HE) stained sections for better orientation. Note the high expression of TRF2 in neurons independent from the brain section. Sale bars indicate 50µm.

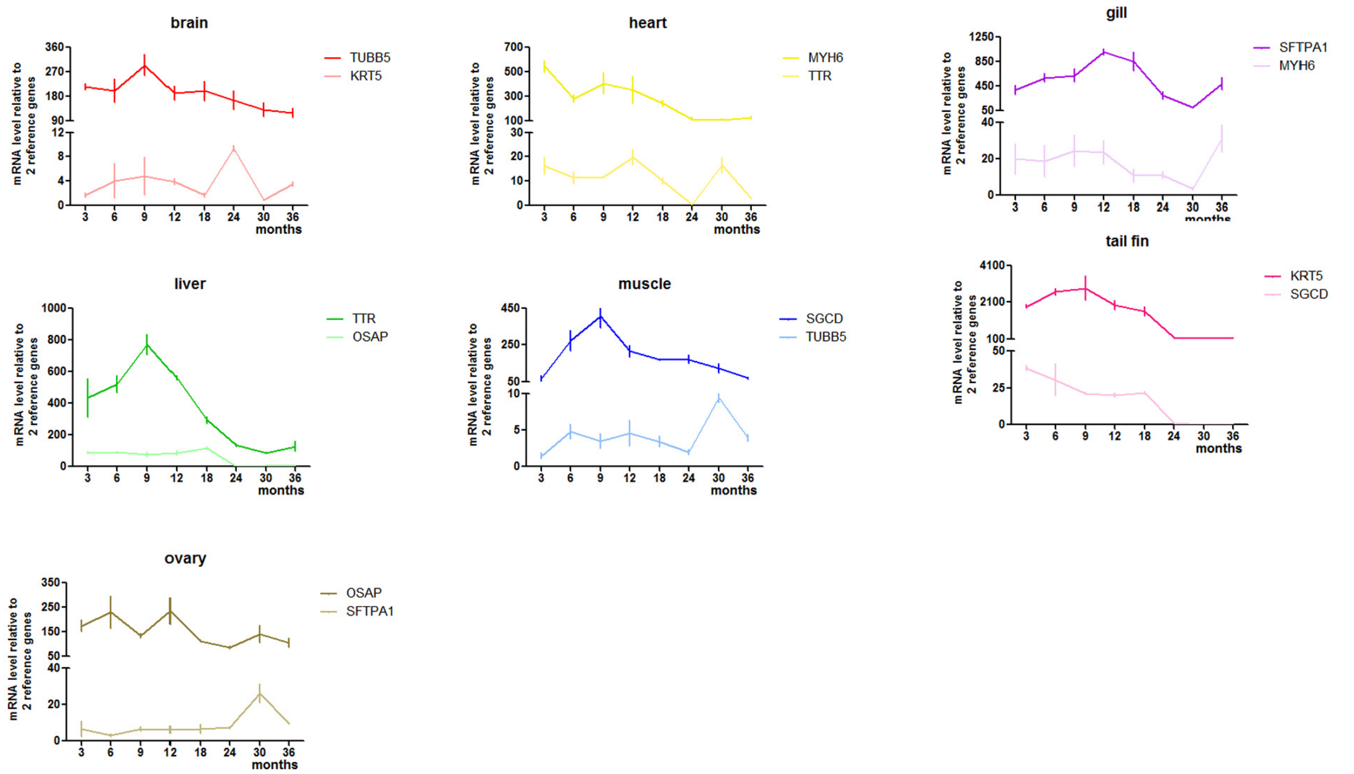

Figure S2 related to Figure 4. Expression of tissues specific markers in the dissected organs from zebrafishes at different ages. Quantitative RT-PCRs for different zebrafish organs was test by different tissues specific primers, including brain, heart, liver, intestine, muscle, gill, tail and ovary from 3 month to 36 month (N=6 each). Dta are presented at mean + SEM.

SUPPLEMENTARY EXPERIMENTAL PROCEDURES

Mice, zebrafish, and tissue preparation

Timed pregnant mice (NMRI) were purchased from Janvier Labs (Le Genest-Saint-Isle, France). Pregnant mice were killed using cervical dislocation, the cerebral wall at early development stages (embryonic day 10 (E10), 12, 14, and 16) or cerebral cortex at later developmental stages (E18, postnatal day 1 (P1), P7, P21) or in adults (P100), the heart, the kidney, and the liver were dissected, and tissues were used to prepare RNA. For immunohistochemistry, whole embryos were used up to E18, for later stages, organs were dissected. Timed zebrafish were purchased from Lab of Shanghai Institute of Haematology (Shanghai Jiaotong University affiliated Ruijin Hospital,Shanghai China). The maintenance, breeding and staging were performed as described previously [1]. The zebrafish were anaesthetized, and dissected on ice using ophthalmic forceps. The different tissues (brain, heart, liver, intestine, muscle, gill, tail and ovary) were separated and used to prepare RNA.

Real-time RT-PCR

Total RNA was isolated from organs using the Trizol reagent (Invitrogen). The RNA pellet was dissolved in diethyl pyrocarbonate-treated H2O. First-strand cDNA synthesis was performed with 0.5 µg of total RNA using oligo(dT) primers and Superscript III reverse transcriptase (Invitrogen) for mouse tissues and Superscript I reverse transcriptase (Takara) for zebrafish tissues. One µl of the reaction product was taken for real time RT-PCR amplification (ABI Prism 7000, Applied Biosystems) using a commercial SYBR® Green kit (Eurogentec, Angers, France; and Tiangen, China). Primer sequences are available on request. Expression of each gene was normalized to the respective Gapdh, actin, and Rplp0 expression for mouse tissues and to the respective actin, and Rplp0 expression for zebrafish tissues.

Mouse tissue samples, histology and immunohistology Samples from at least three different animals per time-point were analyzed. Three µm paraffin sections were used for histological and immunohistological procedures. Haematoxylin-Eosin staining was routinely

performed on all tissue samples. For Trf2 immunohistology, after heat-mediated antigen retrieval and quenching of endogenous peroxidase activity, the antigen was detected after antibody application Trf2 (1:100, rabbit monoclonal, #13136, (Cell Signaling Technology) using the EnVisionTM Peroxidase/DAB Detection System from Dako (Trappes, France). Sections were counterstained with Hematoxylin (Sigma). Omission of the first antibody served as a negative control. Additionally, some slides were incubated with an IgG Isotype Control (1:100, rabbit monoclonal, clone SP137, Abcam) as a negative control. Slides were photographed using a slide scanner (Leica Microsystems, Nanterre, France) or an epifluorescence microscope (DMLB, Leica, Germany) connected to a digital camera (Spot RT Slider, Diagnostic Instruments, Scotland).

Cloning and constructs

The full-length cDNA of zebrafish TRF2(Terfa), neurog1 and c-myb was cloned into the pCS2+ vector. The EcoR I and Xho I enzymatic sites were incorporated into the end of cDNA sequence to facilitate the directional cloning. The following primers were used:
TRF2(Terfa): F:ATGAGCGACAAACCCTGCGAA; R: GACCATCTTGAGCTTGACCAT
Neurog1: F: CCCACCAATAAGGTTATCAA; R: GCAGACTGTCATTAAGGCAAA
C-myb: F: GGGTTGGACCATTGGAAGAA; R: TGTAAAGGCGAGGGTTGATG

Whole-mount in situ hybridization

Whole-mount in situ hybridizations with Digoxigenin- and fluorescein-labeled antisense mRNA probes were transcribed with T3 polymerase from EcoR I-linearized plasmid according to the manufacturer's instructions (Roche). As a control, sense mRNA probes were transcribed from Xho I-linearized plasmid using SP6 polymerase as previously described [2].

Primers used to determine Shelterin expression in zebrafish

TRF1
F- GCTGGAAGAACAGACAGATGTA; R- GAGGCATATTGCTGGTTGAAAG
TRF2
F- CTGCGCCTCATGCAGTTT; R- ATCACCAGCATCTCGCTGAT
TPP1
F- GGAGCACGTCAAGCCATATTA; R- CCACCACCAAACAGACTCAT
POT1
F- CTATCCCTGGTCCTTCTAGTT; R- CTGTAGACCAACCGAATGTG
RAP1
F- AGTTGTGCCGCCTTCTTTA; R- AAGATCTCCACTGGCTTTGAG
TIN2
F- CTGCTGTTCTGGAGGAATGT; R- CCCGAAAGAGGAAGGGAAAG

Primers used to confirm tissue specificity in zebrafish

TUBB5(Tubulin beta 5, brain)
F- GTCAGTGCGGAAACCAAATC; R- AGGCTCCAGATCCACTAGAA
MYH6(Myosin heavy chain 6, heart)
F- GGCACTGAAGACGCAGATAA; R- CTCACCACCATCCAGTTGAA
KRT5(Keratin 5, skin)
F- GACACATCAGTCGTTGTGGA; R- ATCTCAGCCTTGGTGTTACG
TTR(Transthyretin, liver)
F- CATCTCTGTTTGCCCTCTGT; R- ACCAGTCATGTCCACTTTCC
SFTPbb(Surfactant protein Bb, gill)
F- TTCAATGCTGGGACTGTGT; R- TGGCAACAAGCTCTCTACTG
SGCD(Dystrophin-associated glycoprotein, muscle)
F- CCGAGCCGTTCAAAGAGTTA; R- CAGACGTGGGAGCTTGATTT
MGARP(mitochondria-localized glutamic acid-rich protein, ovary)
F- TCCGGTGAGAACATCGTCTA; R- ATGGTTTGGGCTTCCACTC

Primers for housekeeping genes in zebrafish
RPLP0(36b4)

F- TGCTGCTGGCAAACAAAG; R- CACCTTGTCTCCAGGTTTGA
β-actin
F- GGGTATGGAATCTTGCGGTATC; R- CTTCATGGTGGAAGGAGCAA

Morpholino design and microinjection

Morpholino oligonucleotides (MOs) were obtained from Gene Tools (Philomath, OR, USA). The sequence of MOs against TERFA gene was 5’-TTCGCAGGGTTTGTCGCTCATTCTT-3’ which was shown to effectively suppress the expression of TERFA in zebrafish. A standard control MO 5’-TCCACACAGTGGTTCAAATCCCACAT-3’ was also used. The control MO provided by Gene Tools had no target and no significant biological activity. An MO solution was prepared with sterile water and contained

0.1% phenol red as a visualizing indicator. The MO was microinjected into embryos at the 1~2-cell stage with an IM-300 microinjector system (Narishige Scientific Instrument Laboratory, Tokyo, Japan). In preliminary tests, embryos injected with 2 ng of the control MO showed no significant differences in survival rates and morphology compared to WT embryos. Embryos injected with 2 ng of the TERFA MO showed no significant differences in survival rates compared with control MO but had a morphology difference.

Statistical analysis

Data are expressed as means±S.E.M. ANOVA with Bonferroni test as post-hoc test or Mann-Whitney tests were performed as indicated. A P-value of less than 0.05 was considered statistically significant.

SUPPLEMENTARY REFERENCES

1. Kimmel CB, Ballard WW, Kimmel SR, Ullmann B, Schilling TF. Stages of embryonic development of the zebrafish. Dev. Dyn. 1995; 203:253–310.
2. Bennett CM, Kanki JP, Rhodes J, Liu TX, Paw BH, Kieran MW, Langenau DM, Delahaye-Brown A, Zon LI, Fleming MD, Look AT. Myelopoiesis in the zebrafish, Danio rerio. Blood. 2001; 98:643–51.
